# Patient-Derived Explants of Osteoarthritic Synovium as Ex Vivo Model for Preclinical Research

**DOI:** 10.3390/ijms26146665

**Published:** 2025-07-11

**Authors:** Claudia D’Oria, Gilberto Cincinelli, Ramona Bason, Federica Pisati, Francesca Simoncello, Isabella Scotti, Laura Giudice, Ilaria Suardi, Paolo Ferrua, Chiara Fossati, Pietro Simone Randelli, Roberto Caporali, Massimiliano Pagani, Francesca Ingegnoli

**Affiliations:** 1 IFOM ETS—The AIRC Institute of Molecular Oncology, 20122 Milan, Italy; claudia.doria@fht.org (C.D.); ramona.bason@ifom.eu (R.B.); francesca.simoncello@ifom.eu (F.S.); massimiliano.pagani@ifom.eu (M.P.); 2 Rheumatology Clinic, Department of Rheumatology and Medical Sciences, ASST Gaetano Pini-CTO, 20122 Milan, Italy; gilberto.cincinelli@unimi.it (G.C.); isabella.scotti@asst-pini-cto.it (I.S.); laura.giudice1@unimi.it (L.G.); ilaria.suardi@unimi.it (I.S.); 3 Department of Clinical Sciences and Community Health, Dipartimento di Eccellenza 2023–2027, Università degli Studi di Milano, 20122 Milan, Italy; 4 Department of Medical Biotechnology and Translational Medicine, Università degli Studi di Milano, 20122 Milan, Italy; 5 Histopathology Unit, Cogentech, 20122 Milan, Italy; federica.pisati@cogentech.it; 6 Prima Clinica Ortopedica, ASST Gaetano Pini-CTO, 20122 Milan, Italy; paolo.ferrua@unimi.it (P.F.); chiara.fossati@unimi.it (C.F.); pietro.randelli@asst-pini-cto.it (P.S.R.); 7 Department of Biomedical Sciences for Health, Università degli Studi di Milano, 20122 Milan, Italy

**Keywords:** osteoarthritis, synovium, ex vivo model, explants

## Abstract

Osteoarthritis (OA) is the most common chronic arthropathy worldwide. OA synovitis is a common feature that predicts the development and progression of symptoms and joint damage. Although the OA synovium is a target for novel therapies, the development of ex vivo models remains an area requiring further research. We aim to develop a 3D tissue explant culture model of human OA synovium that preserves the architecture and cellular heterogeneity of the original tissue in vitro. We derived tissue explant models from seven patients with OA and followed the culture for up to 10 days, assessing their morphology and cellular composition by immunohistochemistry (IHC) and flow cytometry, respectively. IHC analysis of explant cultures showed that tissue integrity and viability were maintained in our in vitro system. Furthermore, cellular heterogeneity was essentially unchanged when considering CD4^+^ T cells, CD8^+^ T cells, and myeloid fractions in our model. No significant variation was observed in the CD90^+^ and CD90^-^CD55^+^ fractions, which also maintained an activated state as indicated by high levels of FAP expression. An ex vivo OA synovial tissue explant model can maintain pathological tissue integrity for 10 days in culture. This simple and reliable culture system may be useful for analyzing the pathogenesis of OA disease and for the development and testing of therapeutic drugs.

## 1. Introduction

Osteoarthritis (OA) is the most common form of chronic arthropathy characterized by the degeneration of all the tissues in the diarthrodial joints, resulting in significant disability and socioeconomic costs worldwide [1]. OA is a highly heterogeneous disease, and nowadays, despite progress in understanding its pathogenesis, there is still no appropriate therapy nor reliable biomarkers for this disease. Distinct OA phenotypes have been described based on pathobiological mechanisms and structural and functional involvement to help stratify patients in clinical trials [2].

OA affects the whole joint with cartilage and bone damage, low-grade synovial inflammation (synovitis), and fibrosis. Among the joint structures involved in the inflammatory phenotype of OA, the synovium has been shown to contribute to disease progression even in the early stages [3,4,5]. In OA synovium, macrophages and fibroblast-like synoviocytes (FLSs) release pro-inflammatory cytokines, chemokines, and proteolytic enzymes (e.g., matrix metalloproteinases and aggrecans, including ADAMTS4 and ADAMTS5), thereby contributing to the progression of cartilage and whole-joint damage [6,7].

OA synovitis is directly related to chronic joint pain, which is the main symptom of OA and involves both peripheral and central sensitization [3].

OA synovitis is a common feature that can predict the development and progression of symptoms and joint damage [3,4,8]. Although OA synovium is a rational target for preclinical studies testing potential therapeutic approaches, only a limited number of studies have described ex vivo models for osteoarthritic synovium, some of which have used co-culture models [9,10,11,12]; the development of ex vivo models is still an area of unmet research need. From this perspective, we will describe an ex vivo model of human OA synovial explant culture in which we will characterize tissue integrity over time.

## 2. Results

### 2.1. Patients’ Characteristics

Seven patients with primary OA (three men and four women) were included. The mean age ± SD was 72.1 ± 9.6 years. Five patients (71.4%) underwent total knee replacement, and two (28.6%) underwent hip replacement. A radiographic score for OA severity (Kellgren–Lawrence, KL) was obtained through pre-operative, routine radiographs of the affected site, with a median (Q25–Q75) KL of 4 (3–4). The median (Q25–Q75) scores for EQ-5D-pain 1-5 and EQ-5D-movement 1–5 dimensions were 3 (3–3) and 2 (2–3.5), respectively. The median (Q25–Q75) HAQ-DI was 0.875 (0.5–1.063), and the median (Q25–Q75) GH0-100 was 70 (55–72.5). These data are reported in Table 1.

### 2.2. Maintenance of Histologic Features and Viability in OA in Tissue Explant Culture

**Figure 1:**
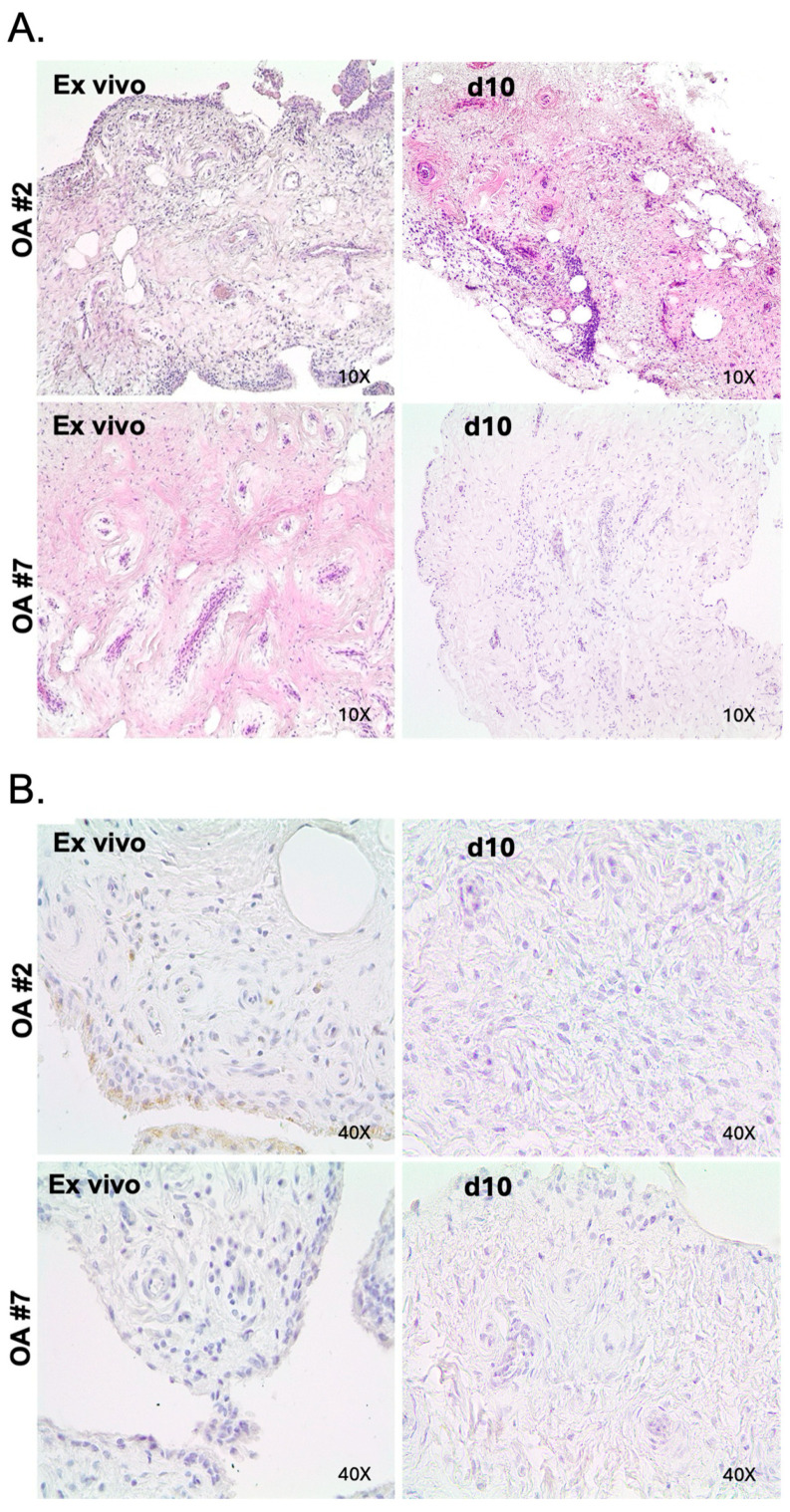
Osteoarthritis tissue explant cultures maintain both (**A**) the histologic features as shown by hematoxylin and eosin-stained sections, and (**B**) the viability as demonstrated by immunohistochemical staining for cleaved caspase 3. Below are two representative patient-derived tissue explants cultured for 10 days (thickness of slices: 4 μm).

To assess the preservation of tissue architecture in our ex vivo model, we performed H&E staining on serial sections from each patient. Figure 1A shows that our explants can maintain the same structure as the ex vivo specimens for up to 10 days in culture.

We also performed immunohistochemical analysis to assess the viability of our tissue culture during this time. As shown in Figure 1B, cleaved caspase-3 staining did not detect any sensitive changes in the viability of our samples compared to the corresponding ex vivo tissues after 10 days in culture.

### 2.3. Maintenance of Cellular Composition in OA in Tissue Explant Culture

**Figure 2:**
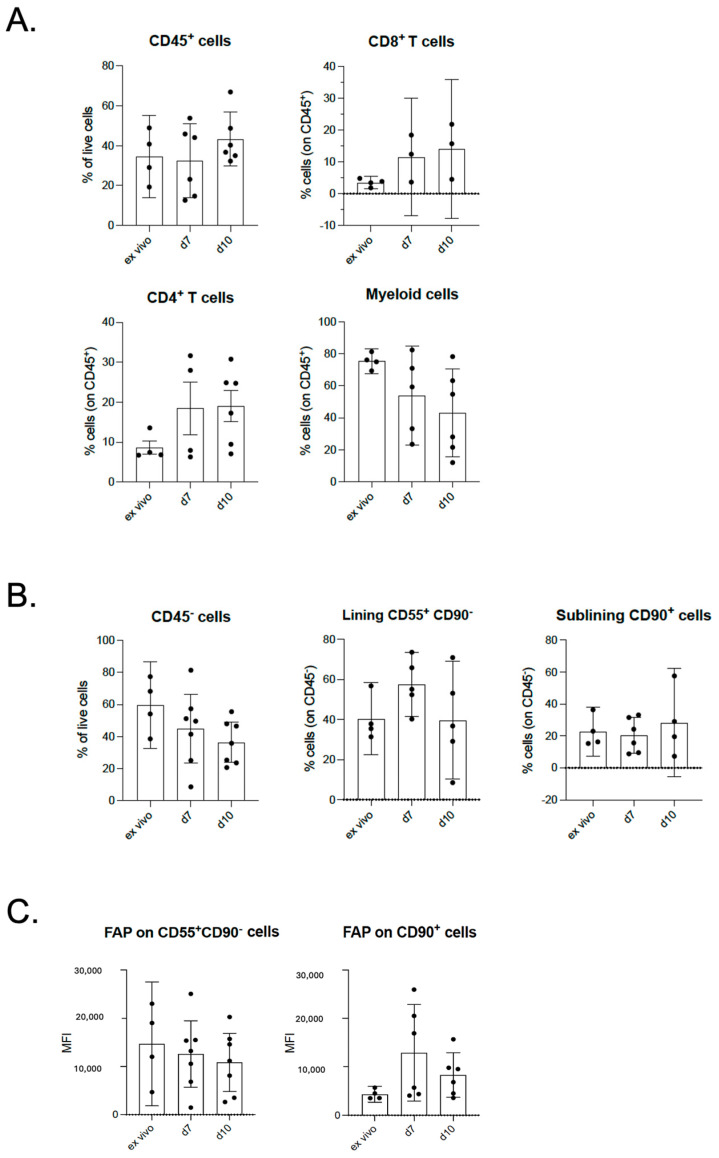
Flow cytometry analysis of different cellular components in osteoarthritis tissue explant at different time points: (**A**) CD8^+^, CD4^+^ T cells, and myeloid compartment; (**B**) sub-lining (CD45^-^CD90^+^) and lining (CD45^-^CD90^-^CD55^+^) synoviocytes; and (**C**) FAP levels in sub-lining and lining synoviocytes expressed as MFI (median fluorescence intensity). Values are expressed as mean with 95% CI (confidence interval).

We assessed the cellular composition in our tissue explant over time by flow cytometry analysis. As shown in Figure 2A, we did not observe a significant decrease in the percentage of CD8^+^ (*p* = 0.3802) and CD4^+^ T cells (*p* = 0.2752), and in the myeloid compartments (N > 4) (*p* = 0.2201). When we analyzed the CD45^-^ fraction in our tissue explant, we observed the persistence of CD90^+^ cells (corresponding to sub-lining synoviocytes) (N > 4) and the CD55^+^CD90^-^ subset (corresponding to lining fibroblasts) (N > 4) after 10 days in culture (Figure 2B). Moreover, when we analyzed the expression of FAP, a marker of fibroblast activation, we detected only a slight (but not significant) decrease in the level of its expression in these two cell subtypes, at least up to 10 days, indicating that these cells are still active and viable (Figure 2C).

## 3. Discussion

Tissue models are important in vitro tools used to study tissue biology, test drugs, and translate knowledge of disease pathogenesis. Several techniques are available for the development and use of tissue models, with varying degrees of feasibility and ability to reproduce tissue characteristics [13]. Most are based on the culture of tissue pieces for short periods of time (usually up to 72 h) and are generated from resections from rheumatoid arthritis patients [14,15].

Some models have been described in OA. The first, developed by Beekhuizen and colleagues [10], focuses on detecting the presence of CD3^+^ cells and CD68^+^ cells up to 21 days, without any quantification during the culture period, another reference to the CD45- compartment (synoviocytes). Other co-culture models have been described, including between a meniscus and synovial membrane (for 7 days) by Favero and colleagues [9] to evaluate the production of inflammatory molecules, and between an osteochondral–synovial membrane (up to 21 days) by Haltmayer and colleagues [10]. Finally, the model by Chan and colleagues [12] describes a co-culture model between synovium and cartilage (not synovium alone) in OA for 7 days, focusing only on detecting the presence of the CD14^+^ and CD90^+^ populations without quantification during the culture period.

In this study, although we are aware of this system’s limitations regarding sample size and full pathological characterization, our model appears to replicate the structure of ex vivo samples macroscopically, without evident aberrations due to culture. Further morphological characterizations are obviously needed. Future studies must address these critical methodological limitations to enhance model robustness and applicability. However, considering our preliminary data, we are quite confident that an in-depth analysis will confirm our observations. Notably, no significant differences were observed in the stromal, CD4^+^, CD8^+^, or myeloid compartments. These results suggest that our system can preserve the main synovial cell populations over a relatively long period. In fact, we were able to maintain most of the cell compartments present in the ex vivo tissue, both immunological and stromal, over a relatively long culture period. Therefore, this system potentially represents a powerful tool to study the crosstalk between all the major components of the synovial tissue without altering their spatial interaction, closely mimicking the in vivo situation. In addition, the addition of specific cytokines to the culture conditions could improve the maintenance of specific cell subsets of interest for longer than 10 days, which is necessary for some types of studies. Our system was intended to be used as a platform for cytotoxicity testing and drug screening experiments that generally require a short period of time (24–72 h). Our 10-day culture model may provide an alternative for longer pharmacological studies, allowing the maintenance of a stable cellular composition and heterogeneity. 

Although OA is a degenerative disease that ultimately leads to joint failure through a process of progressive chondral loss and subchondral bone damage, all articular structures are involved in the pathogenesis to varying degrees. In addition, different phenotyping strategies for OA based on different pathogenic mechanisms are beginning to emerge [16], allowing for a deeper characterization along a disease spectrum that has long been considered monistic.

An inflammatory phenotype of OA has also been described. Low-grade synovitis is the main feature of this phenotype [17]. This subset of OA is particularly evident in some patients with erosive hand [18] and knee OA. Synovitis is not only the phenotypic hallmark but also has prognostic value as it may precede OA and be associated with pain and structural progression [4,19]. In addition, pro-inflammatory cytokines, degradative enzymes, and subsequent synovitis are mechanistically linked to known risk factors for the development of OA, such as trauma, excessive mechanical stress, and obesity [3,20,21].

For these reasons, synovial inflammation and changes associated with specific OA phenotypes are considered an appropriate therapeutic target, but trials using anti-TNF [22] and anti-IL-1 have shown only weak, inconsistent results in terms of pain reduction, MRI synovitis scores, and structural progression in patients with joint inflammation associated with OA. The reason for such drug failure remains largely unknown, but it is plausible that although the abnormal production of pro-inflammatory cytokines has long been documented in OA synovium, it may occur to a lesser extent than in rheumatoid arthritis synovitis [23]. In addition, many cellular and molecular mechanisms have been identified as drivers of OA synovitis [3], which only partially overlap with RA synovitis. These findings would justify disease-specific differences between OA synovitis and other conditions related to synovial inflammation, which are crucial for the implementation of therapeutic strategies, at least for certain subgroups of inflammatory OA.

## 4. Materials and Methods

### 4.1. Study Population

Discarded synovial tissue was obtained from total knee or hip arthroplasties in patients with primary OA (Comitato Etico Milano Area 2, 963_2019). Demographics and questionnaires regarding health-related quality of life (EQ-5D), disability (Health Assessment Questionnaire-Disability Index, HAQ-DI), and global health (GH) assessment were collected from all the patients enrolled.

### 4.2. Synovial Explant Culture

For tissue explant culture, samples were cut into small pieces (~0.3 cm^3^). Each piece was embedded in BME Cultrex matrix (R&D, Pawtucket, RI, USA, cat. #3533-010-02), put on a culture insert of a transwell (pore size 0.4 μm), and then placed in a 24-well plate. The wells were filled with culture medium Dulbecco’s modified Eagle’s medium (Euroclone, Pero (Milan), Italy, # ECM0728L) with 10% fetal bovine serum (Euroclone, Pero (Milan), Italy, # ECS0180L), 500 units/mL penicillin, and 500 μg/mL streptomycin (Euroclone, Pero (Milan), Italy, # ECB3001D) to cover all the tissue lying in the upper part of the insert. The plates were incubated at 37 °C with 5% CO_2_, and the culture medium was exchanged with the same volume of fresh medium every 3 days. The explant model was maintained in culture up to 10 days.

### 4.3. Immunohistochemical Analysis

For histopathological analyses, human samples were fixed in 4% PFA and processed by a Diapath automatic processor as follows. Tissues were dehydrated in graded alcohol (70%, 95%, and 99%), cleared with xylene, and finally immersed in paraffin. Samples were embedded in a paraffin block and stored at room temperature until they were ready to be sectioned.

For HE staining, paraffin was removed with xylene, and the sections were rehydrated in graded alcohol. Tissue samples were incubated in Mayer’s Hematoxylin solution (1 min, RT), rinsed in distilled water, and then immersed in eosin solution (5 s at RT). After dehydration with 80%, 95%, and 100% ethanol (1 min each at RT), the slides were cleared twice in xylene for 5 min and then mounted with mounting medium (DPX).

For Caspase expression, paraffin was removed with xylene, and the sections were rehydrated in graded alcohol. Antigen retrieval was carried out using a preheated target retrieval solution (pH 6.0) for 30 min. Unspecific antigen blocking was performed using a solution with 2% FBS serum and 1% BSA in PBS for 60 min, followed by overnight incubation with the primary antibody diluted 1:100 (Cell signaling, Pero (Milan), Italy, # 9664). Antibody binding was detected using a polymer detection kit (MACH1 Universal HRP Kit, Biocare, Milan, Italy) for 1h at RT, followed by a diaminobenzidine chromogen reaction (Peroxidase substrate kit, DAB, SK-4100; Vector Lab, Newark, NJ, USA) for 2 min at RT. All sections were counterstained with Mayer’s hematoxylin (30 s), dehydrated in graded alcohol, mounted in DPX, and visualized using a bright-field microscope (LEICA, Wetzlar, Germany, DM750).

### 4.4. Flow Cytometric Analysis

**Figure 3:**
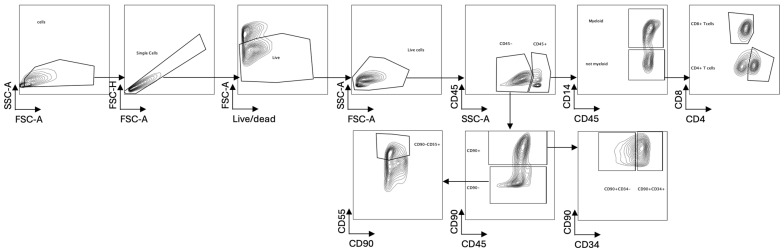
The gating strategy applied for cell subset identification in flow cytometry analysis.

The cultured synovial explants were treated with 1mg/mL of collagenase D at 37 °C in a shaker for 1 h. Samples were stained with Fixable Viability Stain 780 (FVS780-BD HORIZONTM, Milpitas, CA, USA, 565388), according to manufacturer’s instructions, and then with the following extracellular antibodies: anti-human CD45 BUV496 (BD Biosciences, Franklin Lakes, NJ, USA, # 750179), anti-human CD4 Spark YG58 (Biolegend, San Diego, CA, USA, # 344670), anti-human CD8 BUV737 (BD Biosciences # 749367), anti-human CD14 spark nir 685 (Biolegend # 399210), BV421 anti-human CD90 (BD Biosciences # 562556), BV510 anti-human CD55 (BD Biosciences # 742678), and anti-human FAP PE (R&D systems, Minneapolis, MN, USA, # FAB3715P-100). Samples were fixed for 30′ at 4 °C using a fixation/permeabilization kit (BD Biosciences # 554714) and acquired using the Cytek^®^ Aurora instrument, Fremont, CA, USA, following the manufacturer’s instructions (see Figure 3 for gating strategy).

### 4.5. Statistical Analysis

Statistical significance was determined using a mixed-effect analysis, with Tukey’s multiple comparisons test. GraphPad Prism version 9 for Windows, GraphPad Software, San Diego, CA, USA (https://www.graphpad.com/), was used for analysis. *p* values less than 0.05 were considered significant.

## 5. Conclusions

In conclusion, we demonstrated that tissue explant cultures represent a reliable method to replicate OA synovial features. The possibility to reduplicate and manipulate the synovial tissue of OA patients may be determinant for the aim of a deeper, tissue-centered characterization of OA phenotypes, a better definition of disease mechanisms and therapeutic targets, and the application of precision medicine strategies.

## Figures and Tables

**Table 1:** Baseline clinical and demographic characteristics of patients with osteoarthritis included in the analysis.

ID	Age	Gender	Joint Replacement	EQ-5D Pain (1–5)	EQ-5D Movement (1–5)	KL Score (0–4)	HAQ-DI	GH 0–100
158	74	M	Left knee	2	4	4	1.125	40
159	86	F	Right knee	3	3	3	0.375	70
160	67	F	Left hip	3	2	3	0.125	60
161	67	M	Right knee	3	2	4	1.125	50
162	83	M	Right hip	3	4	3	0.625	75
163	59	F	Left knee	3	2	4	0.75	80
164	69	F	Right knee	5	2	4	1	70

M: male; F: female; KL: Kellgren–Lawrence; HAQ-DI: Health Assessment Questionnaire-Disability Index; GH: global health.

## Data Availability

Data may be made available upon reasonable request after publication and after confirming that ethical approval has been obtained.

## Abbreviations

The following abbreviations are used in this manuscript:

OA	Osteoarthritis
FLS	Fibroblast-like synoviocyte
EQ-5D	Questionnaires regarding health-related quality of life
HAQ	Health Assessment Questionnaire
GH	Global health
H&E	Hematoxylin and eosin
IHC	Immunohistochemical
KL	Kellgren–Lawrence
